# Enhancement of RNA-directed DNA methylation of a transgene by simultaneously downregulating a *ROS1* ortholog using a virus vector in *Nicotiana benthamiana*

**DOI:** 10.3389/fgene.2013.00044

**Published:** 2013-04-02

**Authors:** Shungo Otagaki, Megumi Kasai, Chikara Masuta, Akira Kanazawa

**Affiliations:** Research Faculty of Agriculture, Hokkaido UniversitySapporo, Japan

**Keywords:** *Cucumber mosaic virus*, DNA demethylation, RNA-directed DNA methylation, ROS1, virus-induced gene silencing

## Abstract

Cytosine methylation can be induced by double-stranded RNAs through the RNA-directed DNA methylation (RdDM) pathway. A DNA glycosylase REPRESSOR OF SILENCING 1 (ROS1) participates in DNA demethylation in *Arabidopsis* and may possibly counteract RdDM. Here, we isolated an ortholog of *ROS1* (*NbROS1*) from *Nicotiana benthamiana* and examined the antagonistic activity of NbROS1 against virus-induced RdDM by simultaneously inducing RdDM and *NbROS1* knockdown using a vector based on *Cucumber mosaic virus*. Plants were inoculated with a virus that contained a portion of the *Cauliflower mosaic virus* 35S promoter, which induced RdDM of the promoter integrated in the plant genome and transcriptional silencing of the green fluorescent protein gene driven by the promoter. Plants were also inoculated with a virus that contained a portion of *NbROS1*, which induced downregulation of *NbROS1*. Simultaneous induction of RdDM and *NbROS1* knockdown resulted in an increase in the level of cytosine methylation of the target promoter. These results provide evidence for the presence of antagonistic activity of NbROS1 against virus-induced RdDM and suggest that the simultaneous induction of promoter-targeting RdDM and *NbROS1 *knockdown by a virus vector is useful as a tool to enhance targeted DNA methylation.

## INTRODUCTION

Nucleotide-sequence-specific interactions mediated by RNA have a role in the control of gene expression via diverse pathways of RNA silencing, which involve either RNA-guided RNA degradation or epigenetic modification of the genome (Baulcombe, 2004; Brodersen and Voinnet, 2006). RNA-guided epigenetic modification was first discovered in transgenic tobacco plants, in which a viroid complementary DNA (cDNA) integrated in the genome was methylated *de novo* when the plants were infected with the viroid consisting of a self-replicating RNA, a process coined RNA-directed DNA methylation (RdDM; Wassenegger et al., 1994). RdDM can also be induced by viral RNAs (Jones et al., 1998) and transgene-derived RNAs (Mette et al., 2000). In *Arabidopsis*, 24-nt short interfering RNAs (siRNAs) generated through processing of double-stranded RNAs (dsRNAs) with Dicer-like (DCL) 3 act as a mobile signal and direct RdDM (Molnar et al., 2010). When dsRNAs corresponding to a gene promoter are synthesized, RdDM of the promoter and transcriptional gene silencing (TGS) can be induced. Such promoter-targeted gene silencing has been used to modify gene expression in plants (Jones et al., 2001; Sijen et al., 2001; Cigan et al., 2005; Heilersig et al., 2006; Okano et al., 2008; Kanazawa et al., 2011a,b; Otagaki et al., 2011; Kasai and Kanazawa, 2013) and other organisms (Hawkins and Morris, 2008; Suzuki and Kelleher, 2009).

RdDM induces *de novo* methylation of cytosine in all sequence contexts (CG, CHG, and CHH, where H is A, C, or T) at the region of siRNA–DNA sequence homology (Matzke et al., 2009). Factors involved in RdDM and TGS have been identified by analyzing mutants of *Arabidopsis* that are defective in the process. These factors are the canonical RNA silencing machinery that includes DCL and Argonaute (AGO) family proteins, two plant-specific RNA polymerases, Pol IV and Pol V, DOMAINS REARRANGED METHYLTRANSFERASES (DRM1 and DRM2), chromatin-remodeling factors, and several other proteins that can interact with these factors (Matzke et al., 2009; Law and Jacobsen, 2010; Zhang and Zhu, 2011). Cytosine methylation established through RdDM can be maintained through cell division. On the other hand, a family of DNA glycosylases can demethylate cytosine in plants (Chan et al., 2005).

In *Arabidopsis*, DNA glycosylases of the DEMETER (DME) family demethylate cytosine: a family consisting of DME, DME-LIKE proteins DML2 and DML3, and REPRESSOR OF SILENCING 1 (ROS1; Furner and Matzke, 2011). DNA demethylation by DME occurs during reproductive development and is required for genomic imprinting and seed viability (Choi et al., 2002; Gehring et al., 2006), whereas DNA demethylation by ROS1, DML2, and DML3 occurs in vegetative tissues and protects hundreds of loci from potentially deleterious methylation (Penterman et al., 2007; Zhu et al., 2007).

REPRESSOR OF SILENCING 1 was identified in a screen for mutants with deregulated expression of a repetitive transgene comprising the luciferase gene driven by the *RD29A* promoter, in which a low level of siRNAs of the *RD29A *promoter generated from the transgene repeat presumably induces RdDM at both the transgene and endogenous *RD29A* promoters (Gong et al., 2002). In wild-type plants, ROS1 is thought to demethylate DNA, thereby counteracting the RdDM (Gong et al., 2002; Kapoor et al., 2005).

We previously developed a system that induces RdDM using an RNA virus vector (designated CMV-A1) based on *Cucumber mosaic virus* (CMV; Kanazawa et al., 2011a,b; Otagaki et al., 2011). Although dozens of virus vectors have been developed for inducing post-transcriptional gene silencing (PTGS; Kanazawa, 2008; Senthil-Kumar and Mysore, 2011), so far, only the CMV vector efficiently induces TGS of not only transgenes but also endogenous genes (Kanazawa et al., 2011a,b). Such efficient induction of TGS is achieved by the function of the virus-encoded 2b protein, which has the ability to facilitate epigenetic modifications through the transport of siRNAs to the nucleus (Kanazawa et al., 2011a). When transgenic *Nicotiana benthamiana* (line 16c) plants, which express the green fluorescent protein (*GFP*) gene under the control of *Cauliflower mosaic virus* (CaMV) 35S promoter, were infected with recombinant CMVs containing a CaMV 35S promoter segment, the level of *GFP* mRNA decreased and cytosine methylation was induced on the transgene CaMV 35S promoter (Otagaki et al., 2006, 2011). This reduction in *GFP* expression was accompanied by a decrease in RNA polymerase II bound to the CaMV 35S promoter, which indicates the occurrence of transcriptional repression (Kanazawa et al., 2011a). Using this system, we examined the effect of differences in the length and positions of the promoter dsRNA on the induction of RdDM and found that both the length of dsRNA (above a threshold of 81–91 nt) and the frequency of cytosines at symmetric sites (described in Section “Discussion”) in the region targeted by dsRNA were important for the induction of efficient RdDM and heritable TGS (Otagaki et al., 2011).

Assuming that DNA methylation and demethylation act antagonistically at the promoter region targeted by the CMV-A1 vector, a combination of RdDM and inhibition of DNA demethylase function may enhance cytosine methylation at the target DNA. To test this hypothesis, here we isolated an ortholog of *ROS1* in *N. benthamiana* and examined the effect of knocking-down the *ROS1* ortholog on RdDM of the CaMV 35S promoter induced by the virus vector.

## RESULTS

### ISOLATION OF THE *AtROS1* ORTHOLOG FROM *N. benthamiana*

**FIGURE 1:**
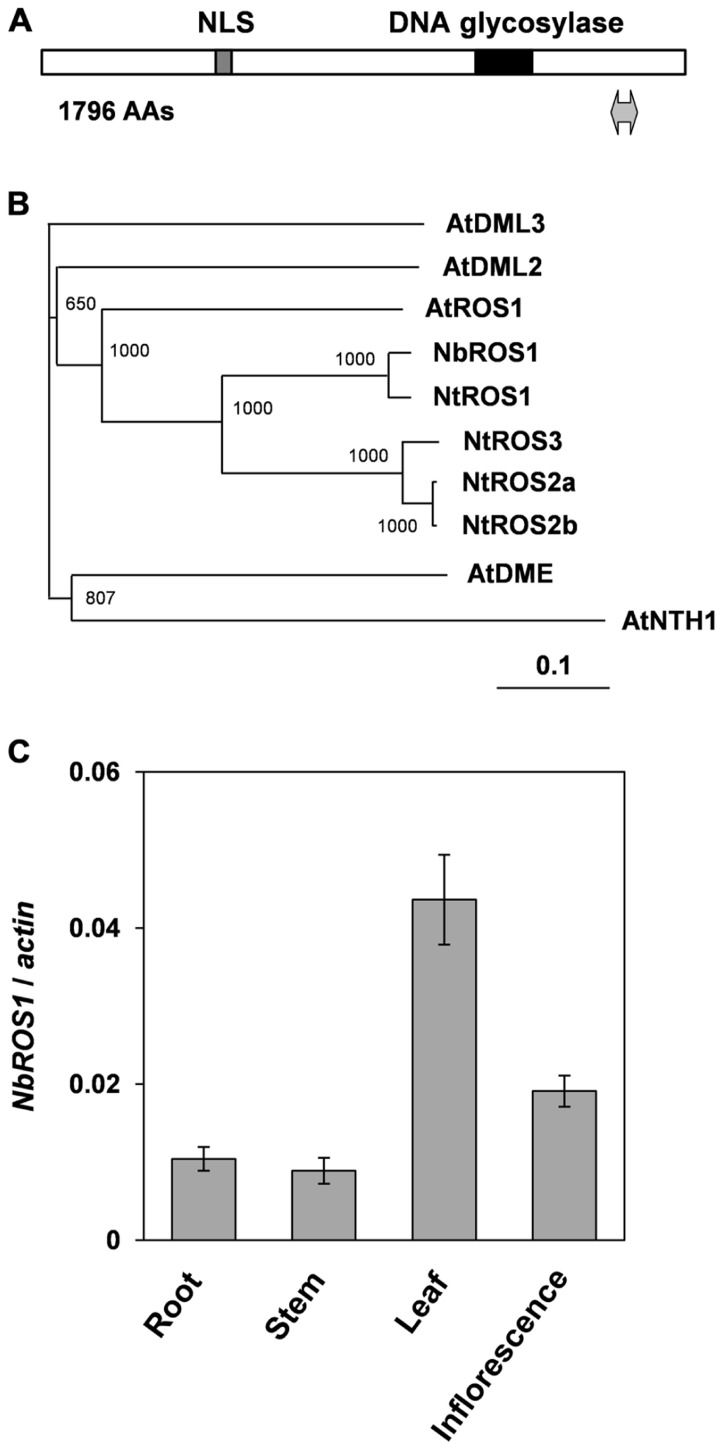
**Characterization of *ROS1* ortholog in *N. benthamiana***. **(A)** Structure of NbROS1 protein. Protein size is shown in number of amino acids (AAs). Hatched and black boxes indicate the nuclear localization signal and DNA glycosylase domain, respectively. The region inserted into the CMV-A1 vector is indicated by a thick line with arrowheads at each end. **(B)** Phylogenetic relationships of DNA glycosylases. The phylogenetic tree was constructed using the neighbor-joining method based on protein sequences deduced from the nucleotide sequences of the genes. Bootstrap values of 1000 replicates are shown. A scale bar represents branch length. Protein sequence of AtNTH1 was used as the outgroup to root the tree. The following sequences were included in the analysis. AtNTH1, *Arabidopsis thaliana* NTH1 (CAC16135); AtDME, *A. thaliana* DEMETER (NP_001078527); AtDML2, *A. thaliana* DEMETER-Like2 (NP_187612); AtDML3, *A. thaliana* DEMETER-Like3 (NP_195132); AtROS1, *A. thaliana* ROS1 (Q9SJQ6); NtROS1, *Nicotiana tabacum* ROS1 (BAF52855); NtROS2a, *N. tabacum* ROS2a (BAF52856); NtROS2b, *N. tabacum* ROS2b (BAF52857); NtROS3, *N. tabacum* ROS3 (BAF52858). **(C)** Expression of *NbROS1*. The *NbROS1* mRNA level relative to the *actin* mRNA level was assessed for root, stem, leaf, and inflorescence tissues. Data are the means and standard errors obtained from three replicates.

Portions of the *ROS1* ortholog in *N. benthamiana* were amplified by reverse transcription-mediated polymerase chain reaction (RT-PCR) using primers designed to anneal regions conserved between *Arabidopsis* *ROS1* and an ortholog of *ROS1* in *N. tabacum* (*NtROS1*). Then a full-length cDNA of *ROS1* ortholog in *N. benthamiana* (designated *NbROS1*) was isolated by RT-PCR in combination with 5′- and 3′-rapid amplification of cDNA ends (RACE) techniques. *NbROS1* was predicted to encode a protein of 1796 amino acids and contained a putative nuclear localization signal and a conserved DNA glycosylase domain (**Figure 1A**). A phylogenetic analysis indicated that NbROS1 was most closely related to NtROS1 among proteins belonging to the DME/ROS1 glycosylase family (**Figure 1B**). We identified two expressed sequence tags (ES887350 and GO612804) that share sequence homology with *NbROS1* by employing the BLASTN search program of the Dana Farber Cancer Institute (DFCI) *N. benthamiana* Gene Index^1^, which indicates that multiple cognate genes are present in the genome. Quantitative RT-PCR using primers that specifically amplified *NbROS1* indicated that the mRNA of *NbROS1* gene was present in root, stem, leaf, and inflorescence (**Figure 1C**), which was consistent with the expression of the *AtROS1* gene in both vegetative and reproductive organs (Gong et al., 2002; Ortega-Galisteo et al., 2008).

### KNOCKDOWN OF *NbROS1* GENE EXPRESSION USING THE CMV-A1 VECTOR

**FIGURE 2:**
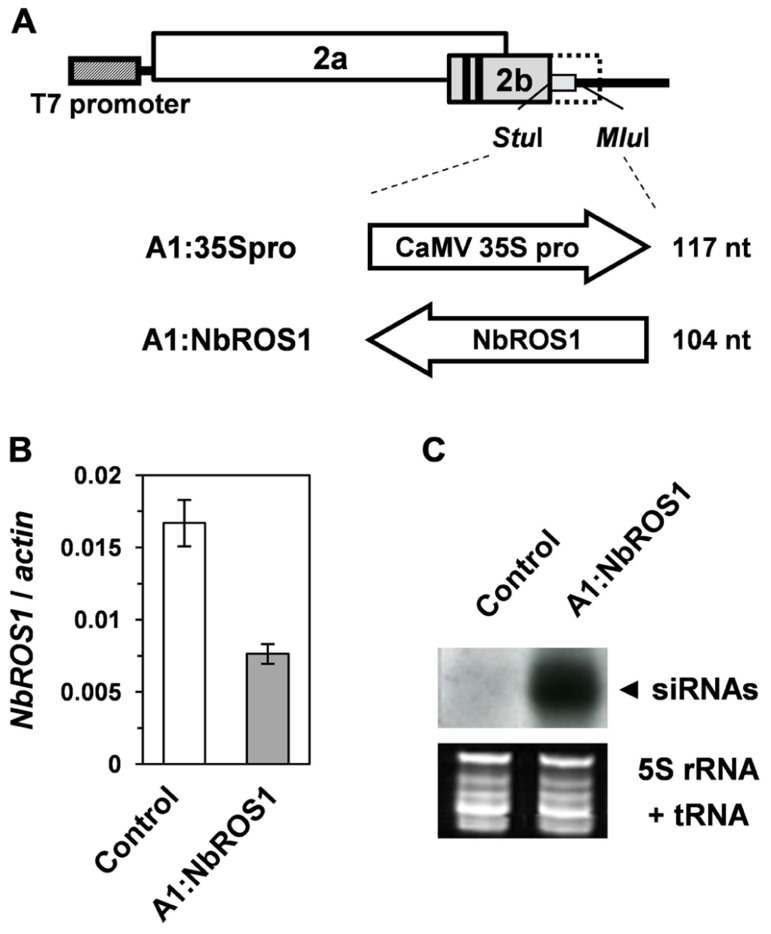
**Gene silencing using the CMV-A1 vector**. **(A)** Schematic representation of the vector constructs targeting the CaMV 35S promoter or the *NbROS1*-coding sequence. **(B)** Changes in mRNA level of *NbROS1* as a consequence of infection with virus that contains the *NbROS1* insert (A1:NbROS1). The *NbROS1* mRNA level was assessed relative to the *actin* mRNA level in leaf tissues at 18 days post-inoculation (DPI). Data are the means and standard errors obtained from three replicates. **(C)** Northern blot analysis of low-molecular weight RNAs isolated from leaf tissues of plants infected with the recombinant CMVs at 18 DPI, probed for the *NbROS1* gene. Ethidium-bromide-stained 5S rRNA and tRNAs bands are shown below the panel to show that an equal amount of the small RNA fraction was loaded. In the experiments in **(B,C)**, both the control and A1:NbROS1-infected plants were infected with A1:35Spro to eliminate non-specific effects of viral infection on the mRNA level of *NbROS1*.

Because of the wide expression of *NbROS1* in tissues including leaves, we induced PTGS of *NbROS1* using a virus vector. A 104-nt portion of the *NbROS1*-coding region (**Figure 1A**) was inserted into the CMV-A1 vector (**Figure 2A**), then *N. benthamiana* plants were infected with the recombinant virus A1:NbROS1. The mRNA level of *NbROS1* decreased (**Figure 2B**) and *NbROS1* siRNAs were produced (**Figure 2C**) in the plants infected with the recombinant virus, which indicated that *NbROS1* was downregulated through PTGS.

### EFFECTS OF *NbROS1* DOWNREGULATION ON TARGETED CYTOSINE METHYLATION INDUCED BY THE VIRUS VECTOR

**FIGURE 3:**
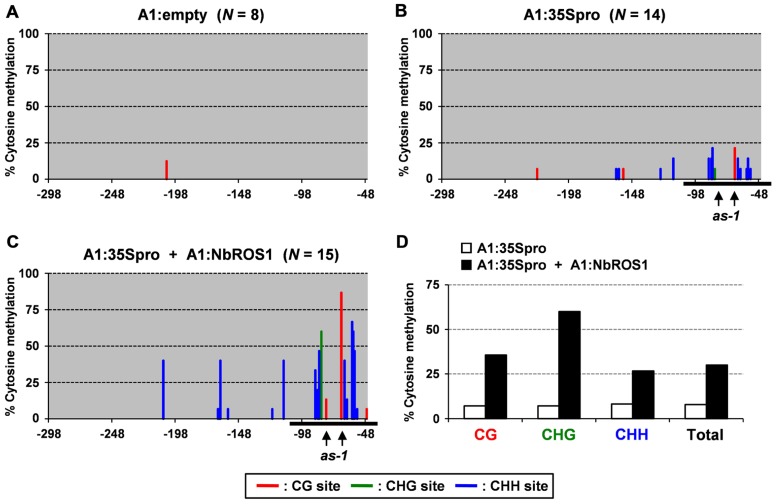
**Cytosine methylation status of CaMV 35S promoter analyzed by bisulfite sequencing**. Cytosine methylation status of the CaMV 35S promoter in plants infected with **(A)** A1:empty that lacked an insert, **(B)** A1:35Spro, and **(C) **both A1:35Spro and A1:NbROS1. The percentages of methylcytosine are shown. Numbers below the line indicate the relative nucleotide positions from the transcriptional start site. Horizontal black lines indicate the targeted region. Red, green, and blue lines indicate frequencies of methylcytosine at the CG, CHG, and CHH sites, respectively. For plants infected with A1:empty, A1:35Spro, and both A1:35Spro and A1:NbROS1, 8, 14, and 15 clones were sequenced, respectively. The positions of two CG sites in the *cis*-acting *as-1* element, to which binding of protein factor(s) is inhibited by cytosine methylation (Kanazawa et al., 2007b), are indicated by vertical arrows. **(D)** Summary of bisulfite sequencing analysis of CaMV 35S promoter in plants infected with A1:35Spro and those infected with both A1:35Spro and A1:NbROS1.

Plants of a transgenic *N. benthamiana* line that carry a transgene containing the CaMV 35S promoter that drives the *GFP* gene in the genome were infected with a virus containing a portion (-116 to +1 region) of the CaMV 35S promoter (“CMV-A1:-116 to +1”; Otagaki et al., 2011). Here we refer to this construct as “A1:35Spro” for convenience (**Figure 2A**). Bisulfite sequencing analysis indicated that infection of plants with this virus induces an increase in cytosine methylation of the promoter via RdDM as previously reported (Otagaki et al., 2011; **Figures 3A,B**).

**Figure A1:**
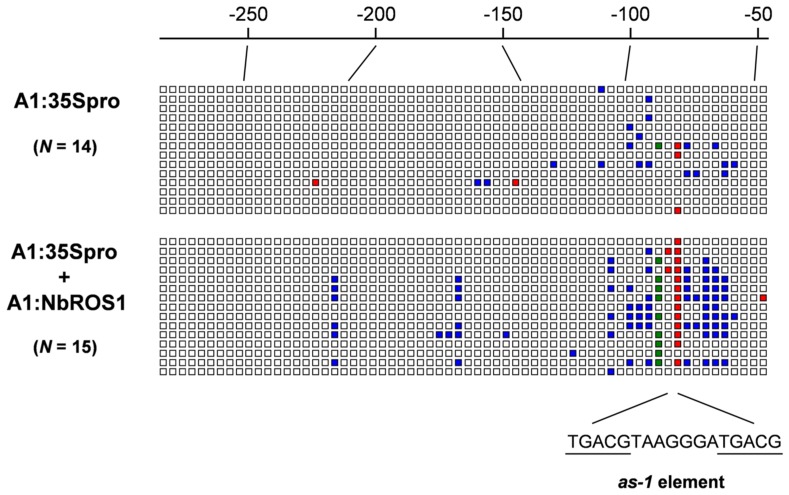
**Graphical representation of cytosine methylation status of CaMV 35S promoter**. Sequencing data for PCR products amplified from bisulfite-treated DNA from plants infected with A1:35Spro (upper) and both A1:35Spro and A1:NbROS1 (lower) have been compiled. For these plants, 14 and 15 clones were sequenced, respectively. Open boxes indicate unmethylated cytosines. Red, green, and blue boxes indicate methylcytosines at the CG, CHG, and CHH sites, respectively. Numbers on the line indicate nucleotide positions relative to the transcription start site of the CaMV 35S promoter. The position of the *cis*-acting *as-1* element, to which binding of protein factor(s) is inhibited by cytosine methylation (Kanazawa et al., 2007b), is shown below the boxes.

We next infected plants with both A1:35Spro and A1:NbROS1 to induce RdDM of the CaMV 35S promoter and downregulation of *NbROS1*, respectively. We found that the level of cytosine methylation in the CaMV 35S promoter was much higher in plants infected with both A1:35Spro and A1:NbROS1 than in plants infected with A1:35Spro alone (**Figures 3B,C**; **Figure A1** in Appendix). Cytosine methylation of the CaMV 35S promoter increased in all sequence contexts, namely, CG, CHG, and CHH (**Figure 3D**).

**FIGURE 4:**
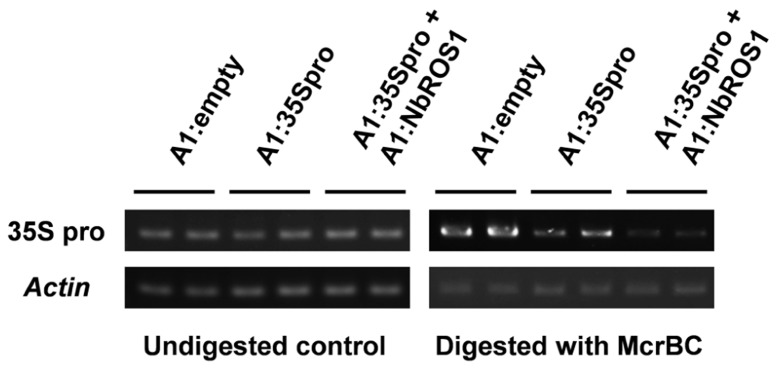
**Cytosine methylation status of CaMV 35S promoter analyzed by McrBC-PCR**. Genomic DNA from plants infected with A1:empty, A1:35Spro, and both A1:35Spro and A1:NbROS1 was digested with McrBC, and was used as a template for amplification of CaMV 35S promoter by PCR. Amplification from undigested DNA and amplification of the *actin* gene were done as controls.

Changes in the frequency of cytosine methylation were also analyzed by a method involving digestion of genomic DNA with a methylation-dependent endonuclease followed by PCR amplification (**Figure 4**). The CaMV 35S promoter region was amplified by PCR from genomic DNA treated with McrBC, an endonuclease that cleaves DNA containing at least two methylcytosines that are preceded by a purine nucleotide and separated each other by 40–80 nt (Sutherland et al., 1992). The level of amplified products of the CaMV 35S promoter in plants infected with both A1:35Spro and A1:NbROS1 was lower than that in plants infected with A1:35Spro alone, the latter of which was lower than that in plants infected with the vector that lacked an insert. These results suggest that RdDM of the promoter was induced in plants infected with A1:35Spro and was enhanced in plants infected with both A1:35Spro and A1:NbROS1.

**FIGURE 5:**
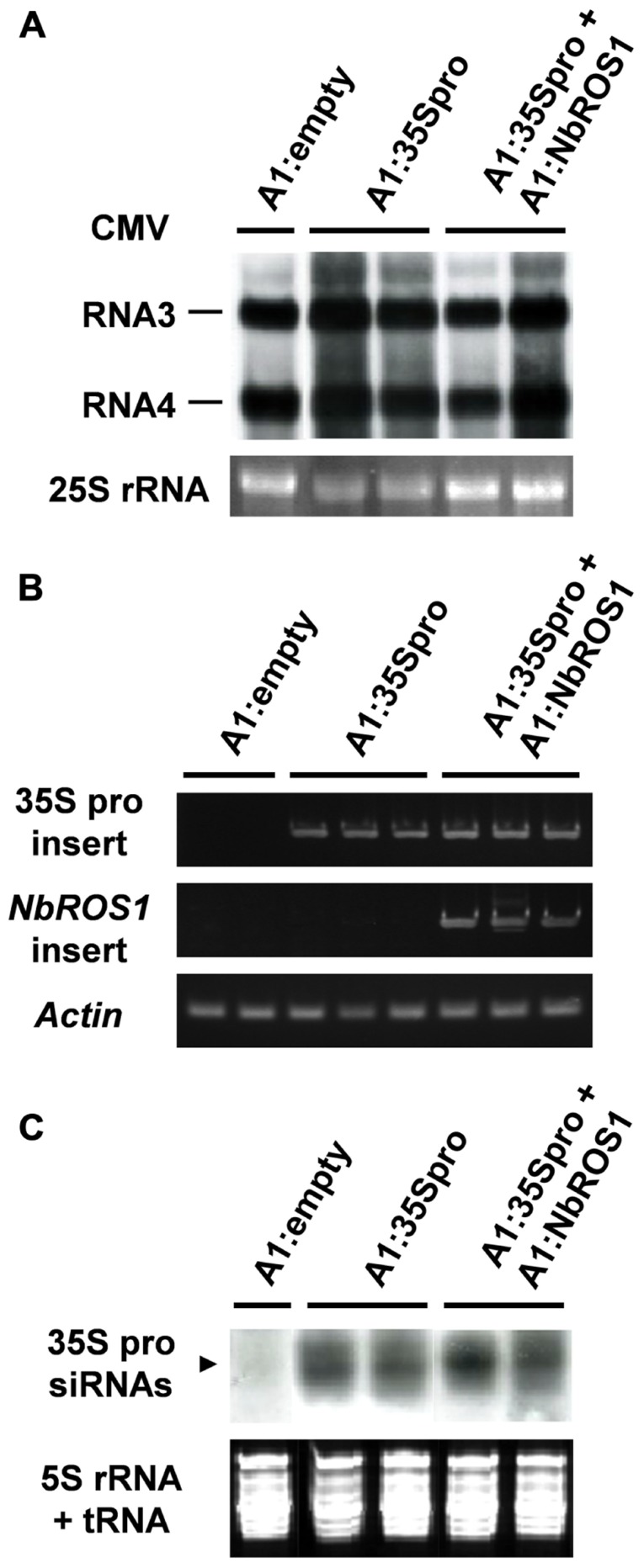
** Analysis of viral RNAs in plants infected with recombinant CMVs**. **(A)** Northern blot analysis of viral RNAs using a CMV RNA-specific probe. Hybridization signals of CMV RNAs 3 and 4 are shown. Ethidium-bromide-stained 25S RNA bands are shown below the panel to show that an equal amount of RNA was loaded. **(B)** RT-PCR analysis of viral RNAs. RT-PCR was done using a combination of primers: a primer that anneals a region adjacent to the cloning site of the viral vector and a primer that anneals the CaMV 35S promoter or *NbROS1* fragment inserted in the vector. A portion of the *actin* gene was amplified as a control. **(C)** Production of siRNAs corresponding to the CaMV 35S promoter in plants infected with recombinant CMVs. Northern blot analysis was done using low-molecular weight RNAs isolated from leaf tissues of the plants infected with the recombinant CMVs at 18 DPI, probed for the CaMV 35S promoter. Ethidium-bromide-stained 5S rRNA and tRNAs bands are shown below the panel to show that an equal amount of the small RNA fraction was loaded. In **(A–C)**, data obtained from two or three individual plants infected with recombinant CMVs are shown.

We analyzed the relative level of viral RNA and production of siRNAs in virus-infected plants. Northern blot analysis indicated that there was no significant difference in the viral accumulation between plants infected with A1:35Spro alone and those infected with both A1:35Spro and A1:NbROS1 (**Figure 5A**). RT-PCR using a primer that anneals a region adjacent to the cloning site of the vector in combination with an insert-specific primer confirmed that the viral RNAs retained the insert segments in the infected plants (**Figure 5B**). In addition, no profound difference was detected in the level of siRNAs corresponding to the CaMV 35S promoter between plants infected with A1:35Spro and those infected with both A1:35Spro and A1:NbROS1 by Northern blot analysis (**Figure 5C**), suggesting that the observed increase in the level of cytosine methylation was not a consequence of a coincidental increase in the level of siRNAs of the targeted promoter by the co-infection. These results suggest that downregulation of *NbROS1* facilitates RdDM of the targeted DNA sequence.

## DISCUSSION

Despite similar substrate specificity, the DME/ROS1 glycosylases have distinct biological roles, with DME functioning during gametogenesis to establish imprinting and the other family members, including ROS1, functioning in vegetative tissues (Law and Jacobsen, 2010). We found that *NbROS1* is expressed in vegetative tissues in *N. benthamiana* as in *Arabidopsis*. We also found that the CaMV 35S promoter of the transgene can be a target of ROS1 as previously observed for the *RD29A* promoter of a transgene (Gong et al., 2002). A recent model for the mechanism(s) of localization of ROS1 protein on a target site involves the sliding of ROS1 protein along DNA (Ponferrada-Marïn et al., 2012), which may fit the notion that ROS1 randomly finds target sites on genomic DNA. The observed demethylation of transgene promoters is consistent with this notion, although whether demethylation by ROS1 occurs at an equal efficiency in different genomic sites is not known.

**FIGURE 6:**
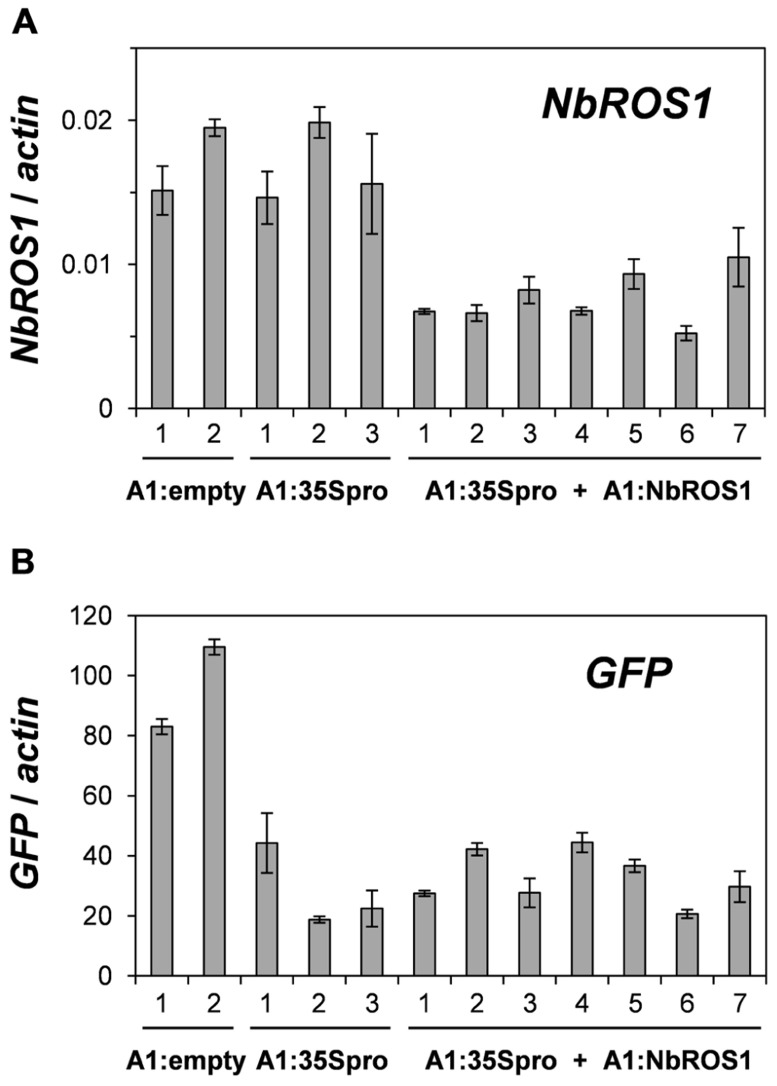
**Changes in mRNA levels of *NbROS1* and *GFP* genes after infection with CMVs containing a portion of CaMV 35S promoter or *NbROS1***. The mRNA levels of *NbROS1* **(A)** and *GFP* **(B)** were analyzed in plants infected with A1:empty, A1:35Spro, or both A1:35Spro and A1:NbROS1. For these plants, data obtained from 2, 3, and 7 individual plants, respectively, are shown. The mRNA levels were assessed relative to the *actin* mRNA level in leaf tissues at 18 DPI. Data are means and standard errors obtained from three replicates.

We have previously reported TGS of the *GFP* gene driven by the CaMV 35S promoter through RdDM of the promoter (Otagaki et al., 2011). The extent of *GFP* TGS was affected by the length and cytosine frequency of the promoter segment inserted in the CMV-A1 vector (Otagaki et al., 2011). Both the extent of *GFP* mRNA reduction and the level of cytosine methylation induced by the CMV-A1:-116 to +1 (A1:35Spro in the present study) was lower than those induced by other constructs of a similarly sized insert (e.g., CMV-A1:-208 to -89; Otagaki et al., 2011). Therefore, we had expected that infection of plants with both A1:35Spro and A1:NbROS1 might result in an increase in both the extent of mRNA reduction and the level of cytosine methylation. However, no profound enhancement of *GFP* mRNA reduction was brought about by the downregulation of *NbROS1* (**Figure 6**), although an increase in the level of cytosine methylation was induced by the treatment (**Figures 3** and **4**). These results suggest that the level of cytosine methylation is not necessary tightly linked with the extent of TGS, although TGS induction was always associated with cytosine methylation of the promoter in this system (Otagaki et al., 2011). We have found that the lower level of TGS induction by CMV-A1:-116 to +1 (A1:35Spro) is correlated not only with a less cytosine methylation but also a lower frequency (3.4/100 nt; 4/117 nt) of cytosines at symmetrical sites (CG and CHG) in the target DNA region: the latter value of the other constructs that induced a higher extent of TGS was 5.4–9.8/100 nt (Otagaki et al., 2011). Taking into account such a constraint in terms of sequence composition, a plausible explanation for the lack of additional effect on TGS is that the extent of mRNA reduction by A1:35Spro was already at a maximum irrespective of the expression level of *NbROS1*. The strategy of co-inoculation with a virus for downregulating a *ROS1* ortholog may be useful for enhancing TGS of the target, particularly when the levels of viral propagation and/or production of promoter siRNAs are limited.

We found that viral RNA levels in plants infected with A1:35Spro were similar to those in plants infected with both A1:35Spro and A1:NbROS1. Despite the co-existence of A1:35Spro with A1:NbROS1, the level of siRNAs derived from A1:35Spro in plants infected with both A1:35Spro and A1:NbROS1 was not different from that in plants infected with A1:35Spro alone (**Figure 5C**). In this regard, the rate of degradation of A1:NbROS1 RNA may be higher than that of A1:35Spro RNA because degradation of A1:NbROS1 RNA can be amplified by *NbROS1* PTGS. This notion is consistent with a previous observation that virus-induced PTGS can lead to elimination of the viral RNA (Ruiz et al., 1998; Vaistij et al., 2002). Although viral RNA levels at different time points remain elusive, the present results suggest that the co-infection does not cause an extensive interference between the viruses, e.g., an enhancement of the degradation of A1:35Spro RNA *in trans* by siRNAs derived from A1:NbROS1 RNA.

In terms of controlling virus-induced changes, the effect of virus-induced PTGS has been enhanced by placing a target gene segment in an inverted repeat position in a viral vector (Lacomme et al., 2003) and was found to be higher in a mutant of a gene that encodes a protein involved in the 3′ end formation of RNA (Herr et al., 2006). To our knowledge, however, no report for enhancing virus-induced RdDM has been published.

The first process that allows the control of the level of targeted DNA methylation may be the induction phase of RdDM, in which accumulation of siRNAs in the nucleus can affect the level of *de novo* methylation. In fact, a transgene-derived hairpin RNA that resides in an intron and thus is expected to be retained in the nucleus was found to efficiently trigger RdDM (Dalakouras et al., 2009). In this regard, the CMV-A1 vector originally has the advantage that the 2b protein encoded in the vector facilitates RdDM through the transport of siRNAs to the nucleus (Kanazawa et al., 2011a). Another process that can be modified involves the maintenance of methylation and/or demethylation, which was tested in this study. Here we found that downregulation of *NbROS1* enhanced virus-induced RdDM, which consequently provides evidence for antagonistic activity of NbROS1 against virus-induced RdDM. Thus, the present method can be used to control the level of cytosine methylation in a targeted DNA region via RdDM.

## MATERIALS AND METHODS

### PLANT MATERIALS

Wild-type *N. benthamiana* plants and plants of transgenic *N. benthamiana* line 16c (Ruiz et al., 1998) were used for analyses. Plants were grown under a 16-h light/8-h dark cycle at 24°C.

### ISOLATION OF THE *NbROS1* GENE

Portions of *NbROS1* were amplified from cDNA by PCR using primers designed to anneal regions conserved between *AtROS1* (DDBJ/EMBL/GenBank accession AY286009) and *NtROS1* (No. AB281587). A cDNA fragment covering the entire coding region of *NbROS1* was isolated using PCR in combination with 5′- and 3′-RACE techniques using a SMART RACE cDNA Amplification Kit (Clonetech) according to the manufacturer’s instructions. Primers used for PCR in this study are listed in **Table A1** in Appendix. The *NbROS1* cDNA sequence data has been deposited in the DDBJ database under accession number AB778815.

### ISOLATION AND ANALYSIS OF RNA

Total RNA was isolated from leaf tissues of *N. benthamiana* plants at 18 days post-inoculation (DPI) as described previously (Otagaki et al., 2006). For analyzing *NbROS1* and *GFP* expression, cDNA synthesis, RT-PCR and quantitative RT-PCR were done as described previously (Kasai et al., 2012). In all PCR experiments, a reaction mixture without reverse transcriptase was used as a control to confirm that no amplification occurred from genomic DNA contaminants in the RNA sample. Amplification of a single DNA species was confirmed by both melting curve analysis of quantitative RT-PCR and gel electrophoresis of PCR products. For analyzing viral RNAs, Northern blot analysis was done as described previously (Otagaki et al., 2006). A CMV RNA-specific probe was prepared by amplifying a portion of the pCY3 plasmid containing the cDNA of CMV-Y RNA 3 (Suzuki et al., 1991). RT-PCR was done as described above except that cDNA was synthesized using a mixture of random 9-nt primers (TaKaRa).

### PHYLOGENETIC ANALYSIS

The protein sequences deduced from the nucleotide sequences of DNA glycosylase genes were aligned using the CLUSTAL W Multiple Sequence Alignment Program version 2.1^2^ (Thompson et al., 1994). A phylogenetic tree was constructed using the neighbor-joining (NJ) method (Saitou and Nei, 1987) based on the protein sequences.

### INOCULATION OF RECOMBINANT VIRUS

For inducing RdDM of the CaMV 35S promoter, the -116 to +1 region (positions are relative to the transcription start site) of the CaMV 35S promoter was amplified by PCR. The amplified fragment was cloned between the *Stu*I and *Mlu*I sites of the CMV-A1 vector as described previously (Otagaki et al., 2011). For the downregulation of *NbROS1*, a 104-bp portion of the *NbROS1*-coding region was also amplified by PCR and the amplified fragment was cloned into the same site in the vector in the antisense orientation. Plasmids containing full-length cDNA of viral RNA were transcribed *in vitro*, and leaves of young *N. benthamiana* plants were dusted with carborundum and rub-inoculated with the transcripts as described previously (Otagaki et al., 2006).

### siRNA DETECTION

Low-molecular weight RNAs were isolated and siRNAs were detected by gel-blot analysis according to the method of Goto et al. (2003). DIG-labeled RNAs corresponding to the -345 to +1 region of the CaMV 35S promoter (Otagaki et al., 2006, 2011) and the 104-bp portion of the *NbROS1*-coding region were used as a probe.

### BISULFITE SEQUENCING ANALYSIS

DNA was isolated from leaf tissues at 24 DPI using the Nucleon PhytoPure DNA extraction kit (GE Healthcare). Bisulfite treatment of DNA and subsequent PCR amplification were done as described previously (Kanazawa et al., 2007a). As a control to ensure that bisulfite treatment was complete, DNA isolated from *Arabidopsis *leaves was simultaneously treated. A region of the *Arabidopsis ASA1* gene that is not methylated was amplified as previously reported (Kanazawa et al., 2007a). All five cloned sequences of PCR products showed complete conversion of cytosines to thymidines.

### McrBC-PCR ANALYSIS

DNA (500 ng) was digested with McrBC for overnight and precipitated with ethanol. After centrifugation, the pellet was dissolved in water. One-fifth volume of the DNA solution was used as a template for amplification by PCR. The products of PCR were analyzed by electrophoresis on an agarose gel.

## Conflict of Interest Statement

The authors declare that the research was conducted in the absence of any commercial or financial relationships that could be construed as a potential conflict of interest.

## Footnotes

1 http://compbio.dfci.harvard.edu/tgi/plant.html

2 http://clustalw.ddbj.nig.ac.jp/top-e.html

## APPENDIX

**Table A1:** List of PCR primers used in the present study.

Target region	Primer name	Primer sequence (5′ to 3′)
**Primers for cloning *NbROS1* cDNA**
*NbROS1*	NtROS1 343F	CCAAGTTTGGCACCAATATG
	NtROS1 768R	CAAGACTTCATCTGCTGGTG
	NtROS1 4206F	TCTTGAATGGCTGAGAGACGTTCC
	NtROS1 4621R	CAAGCCTTGCGCTTGCAAAAGCAC
	NbROS1 5′-RACE R1	CATGCTGCCACATGTTGACCTTCAG
	NbROS1 5′-RACE R2	GTTGCCTATTCCCGCATATTGGTGC
**Primers for quantitative RT-PCR**
*NbROS1*	NbROS1 real 3′ F	CCAAGAAGCTGGTAGGTTAT
	NbROS1 real 3′ R	GCAAACACCTCGTTTAACTT
*GFP*	mGFP5 +148F	ACTGGAAAACTACCTGTTCC
	mGFP5 +344R	TCAAACTTGACTTCAGCACG
*Actin*	Nb-actF	GAAGATACTCACAGAAAGAGG
	Nb-actR2	GGAGCTAATGCAGTAATTTCC
**Primers for cloning DNA fragments into the CMV-A1 vector**
*NbROS1*	StuI NbROS1 anti F	AGGCCTGCCAGAAGATAGGAGCATGG
	MluI NbROS1 anti R	ACGCGTCGGCTCAGAACTGAACATCA
CaMV 35S promoter	StuI 35S –116F	AAGGCCTCTTCAAAGCAAGTGGATTGATG
	MluI 35S +1R	CGACGCGTTCCTCTCCAAATGAAATGAAC
**Primers for bisulfite sequencing analysis**
CaMV 35S promoter	35S –346F bisulfite T	TATTGAGATTTTTTAATAAAGGTAA
	35S +1R bisulfite A	TCCTCTCCAAATAAAATAAACTTC
	35S –323F bisulfite T	TAATATTTGGAAATTTTTTTGGATT
	35S –21R bisulfite A	TTCCTTATATAAAAAAAAAATCTTAC
**Primers for McrBC-PCR analysis**
CaMV 35S promoter	35S –345F	ATTGAGACTTTTCAACAAAGGG
	35S +1R	TCCTCTCCAAATGAAATGAAC
*Actin*	Nb-actF	GAAGATACTCACAGAAAGAGG
	Nb-actR2	GGAGCTAATGCAGTAATTTCC
**Primers for RT-PCR analysis of viral RNAs**
A1:35Spro	RNA2 2327F	ATTCAGATCGTCGTCAGTGC
	35S +1R	TCCTCTCCAAATGAAATGAAC
A1:NbROS1	RNA2 2327F	ATTCAGATCGTCGTCAGTGC
	MluI NbROS1 anti R	ACGCGTCGGCTCAGAACTGAACATCA
*Actin*	Nb-actF2	CATCATGAAGTGTGACGTTG
	Nb-actR2	GGAGCTAATGCAGTAATTTCC
**Primers for preparing probes for Northern blot analysis**
*NbROS1*	StuI NbROS1 anti F	AGGCCTGCCAGAAGATAGGAGCATGG
	MluI NbROS1 anti R	ACGCGTCGGCTCAGAACTGAACATCA
CMV	CMV-DET-5-340	GCGCGTCGACGTTGACGTCGAGCACCAAC
	CMV-DET-3-340	CCATCGATTGGTCTCCTTTTGGAGGCC
CaMV 35S promoter	35S –345F	ATTGAGACTTTTCAACAAAGGG
	35S +1R	TCCTCTCCAAATGAAATGAAC
