# Corrigendum: Enhancement of RNA-directed DNA methylation of a transgene by simultaneously downregulating a *ROS1* ortholog using a virus vector in *Nicotiana benthamiana*

**DOI:** 10.3389/fgene.2016.00021

**Published:** 2016-02-29

**Authors:** Shungo Otagaki, Megumi Kasai, Chikara Masuta, Akira Kanazawa

**Affiliations:** Research Faculty of Agriculture, Hokkaido UniversitySapporo, Japan

**Keywords:** *Cucumber mosaic virus*, DNA demethylation, RNA-directed DNA methylation, ROS1, virus-induced gene silencing

We have become aware that the processing of image data in Figures 5A,C including image assembly was done erroneously in this article. To correct these errors, we prepared a new figure from the original data. There are no associated changes to be made in the main text.

**Figure 5:**
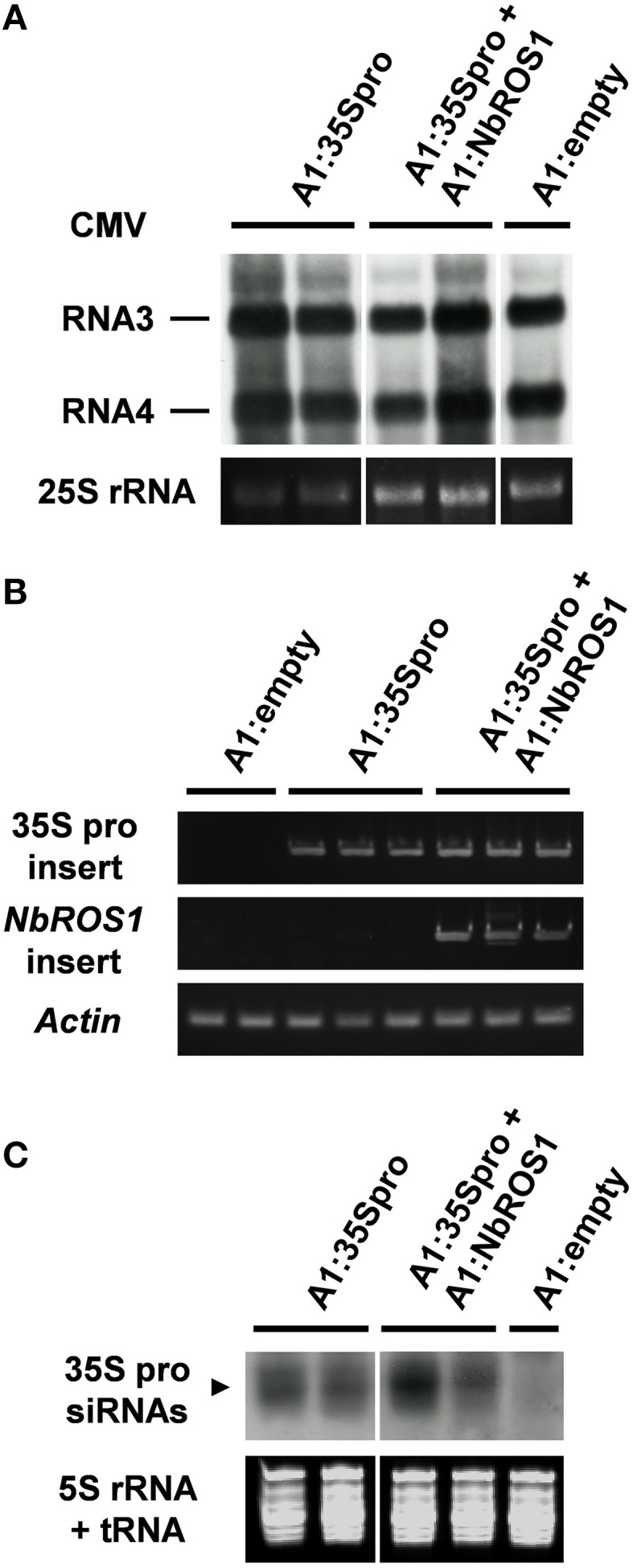
**Analysis of viral RNAs in plants infected with recombinant CMVs. (A)** Northern blot analysis of viral RNAs using a CMV RNA-specific probe. Hybridization signals of CMV RNAs 3 and 4 are shown. Ethidium-bromide-stained 25S RNA bands are shown below the panel to show that an equal amount of RNA was loaded. **(B)** RT-PCR analysis of viral RNAs. RT-PCR was done using a combination of primers: a primer that anneals a region adjacent to the cloning site of the viral vector and a primer that anneals the CaMV 35S promoter or *NbROS1* fragment inserted in the vector. A portion of the *actin* gene was amplified as a control. **(C)** Production of siRNAs corresponding to the CaMV 35S promoter in plants infected with recombinant CMVs. Northern blot analysis was done using low-molecular weight RNAs isolated from leaf tissues of the plants infected with the recombinant CMVs at 18 DPI, probed for the CaMV 35S promoter. Ethidium-bromide-stained 5S rRNA and tRNAs bands are shown below the panel to show that an equal amount of the small RNA fraction was loaded. In **(A–C)**, data obtained from two or three individual plants infected with recombinant CMVs are shown. The images of RNA samples that were not adjacent to each other in the original gel were separated by a line.

## Author contributions

On the basis of the original data obtained by SO, the figure was prepared. All authors approved the manuscript.

### Conflict of Interest Statement

The authors declare that the research was conducted in the absence of any commercial or financial relationships that could be construed as a potential conflict of interest.

