# Expression of Concern: Enhancement of RNA-directed DNA methylation of a transgene by simultaneously downregulating a ROS1 ortholog using a virus vector in *Nicotiana benthamiana*

**DOI:** 10.3389/fgene.2016.00202

**Published:** 2016-11-07

**Authors:** Frontiers in Genetics

With this notice, Frontiers states its concerns about the reliability of some of the findings of the article “Enhancement of RNA-directed DNA methylation of a transgene by simultaneously downregulating a ROS1 ortholog using a virus vector in *Nicotiana benthamiana*” published on 2nd April 2013.

In full accordance with Frontiers procedures, following an investigation directed by our Chief Editors, Dr. David Allison and Dr. Richard Jorgensen, the authors are currently performing further experiments to address these issues.

The situation will be updated as soon as the investigation is complete.

