# Corrigendum: Enhancement of RNA-directed DNA methylation of a transgene by simultaneously downregulating a *ROS1* ortholog using a virus vector in *Nicotiana benthamiana*

**DOI:** 10.3389/fgene.2017.00005

**Published:** 2017-02-01

**Authors:** Shungo Otagaki, Megumi Kasai, Chikara Masuta, Akira Kanazawa

**Affiliations:** Research Faculty of Agriculture, Hokkaido UniversitySapporo, Japan

**Keywords:** *Cucumber mosaic virus*, DNA demethylation, RNA-directed DNA methylation, ROS1, virus-induced gene silencing

We have become aware that an incorrect image was mistakenly used for Figure 2C in the original publication. The correct version of Figure 2 is shown here. We should also note that the primers used for quantitative RT-PCR of the *NbROS1* gene listed in Table A1 turned out to have a sequence mismatch with the target. The mismatched nucleotides in these primers are underlined: 5′-CCAAGAAGCTGGTAGGTTAT-3′ (NbROS1 real 3′ F); 5′-GCAAACACCTCGTTTAACTT-3′ (NbROS1 real 3′ R). We found that a set of primers without sequence mismatch (5′-CCAAGAAGCTGGTAGGCTAT-3′; 5′-GCAAACACCTCGTTAACTT-3′) yielded amplification products at a higher level, although both primer sets amplified a single DNA fragment and were valid for quantification, i.e., comparison of the relative level of *NbROS1* mRNA between samples.

**Figure 2:**
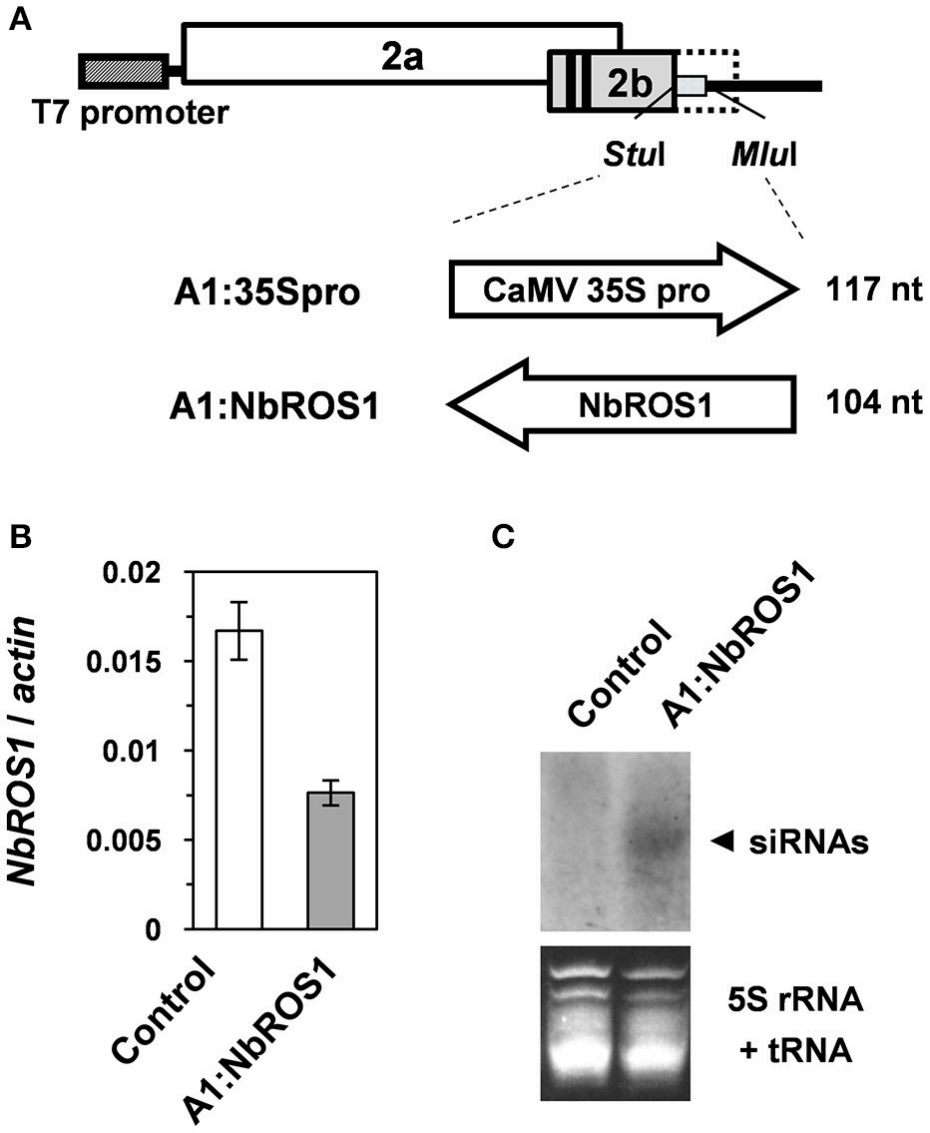
**Gene silencing using the CMV-A1 vector**. **(A)** Schematic representation of the vector constructs targeting the CaMV 35S promoter or the *NbROS1* coding sequence. **(B)** Changes in mRNA level of *NbROS1* as a consequence of infection with virus that contains the *NbROS1* insert (A1:NbROS1). The *NbROS1* mRNA level was assessed relative to the *actin* mRNA level in leaf tissues at 18 days post-inoculation (DPI). Data are the means and standard errors obtained from three replicates. Both the control and A1:NbROS1-infected plants were infected with A1:35Spro to eliminate nonspecific effects of viral infection on the mRNA level of *NbROS1*. **(C)** Northern blot analysis of low-molecular weight RNAs isolated from leaf tissues of plants infected with A1:NbROS1 and the control virus that lacked an insert at 14 DPI, probed for the *NbROS1* gene. Ethidium-bromide-stained 5S rRNA and tRNAs bands are shown below the panel to show that an equal amount of the small RNA fraction was loaded.

The correct Figure 2 with its legend appears below. The authors apologize for any inconvenience caused.

## Conflict of interest statement

The authors declare that the research was conducted in the absence of any commercial or financial relationships that could be construed as a potential conflict of interest.

